# Case of Extensive Wound of the Larynx and Pharynx, Terminating Successfully

**Published:** 1817-11

**Authors:** William Boyd

**Affiliations:** lately Surgeon of the Naval Hospital at Port Mahon, Minorca


*Case of extensive Wound of the Larynx and Pharynx, terminating successfully.* Communicated by WILLIAM BOYD, M. D. lately Surgeon of the Naval Hospital at Port Mahon, Minorca.

THE following case exhibited an instance of recovery contrary to the expectation of every one that saw it; the details, therefore, as taken down at the time, seem worthy of being recorded. The subject of it was a seaman, by name *George Trowhill*, 35 years of age, of a stout make and robust habit, and usually in the enjoyment of good health. On the 21st of December, 1813, he came under my care in the hospital; and his condition, on my first seeing him, is thus accurately described on the Hospital Ticket.

Countenance expressive of the greatest anxiety, distress, and impatience; features shrunk; eyes dull, languid, and inexpressive; at times he rolls them wildly, and stares with a look of agony and distress, which, however, is soon succeeded by the former listless and regardless appearance. He is extremely restless, tossing about in bed, and throwing off the bed-clothes; and will not remain in the position advised for any length of time. Face pale; lips livid, dry, and quivering; extremities cold and tremulous: refuses to answer questions, and seems much irritated when any are put to him.

There is a large incised wound (about four inches in length) on the upper and fore part of the neck, immediately in the space betwixt the *os hyoides* and *thyroid* cartilage, extending completely across from one angle of the lower jaw to the other; the edges of which being laid aside (for its proper examination), the internal wound presents a very formidable appearance. There is an entire separation of the bone and cartilage from each other at their fore and lateral parts, forming a deep ragged wound, in which we observed, with the slightest attention, almost the whole extent of the inferior part of the os hyoides, and superior part of the thyroid cartilage, as also the cavity of the larynx, whence a quantity of frothy mucus, tinged with blood, is constantly thrown out, and drawn in, by each successive expiration and inspiration. There is also a hard convulsive cough, and difficulty of breathing, approaching almost to suffocation. Pulse 100, and almost imperceptible; skin cold and dry; tongue clean; great thirst; other functions natural.

This wound was inflicted by himself about an hour ago, by means of a large clasp knife. By account of the medical gentleman attending, the wound has penetrated the pharynx; for, in endeavouring to drink some water while on board, the liquid flowed through the external wound in a full stream, endangering suffocation. The cause which led him to commit this rash act, was a circumstance of criminality on his own part, which, however, it is not material to state in this report.

The edges of the wound had been brought into contact by means of stitches and adhesive straps, which are now completely displaced by his being so restless.

*Let the wound be brought together by means of adhesive straps; and*

*Let him be placed in the sitting posture, with his head inclined forward, nearly resting upon the breast. To be secured thus by proper bandages.*

*Horâ 2dâ,* P. M. General appearance much as at last report; but he is now less restless, and allows his head to be placed and kept in the position directed. Pulse 108, small and rather hard; temperature 98. Skin dry; thirst urgent: no stool since admission. Respiration has a wheezing sound, something like that attending *croup:* there is a considerable moisture on the dressings, which, on inquiry, I find is from his endeavouring to swallow some liquid; but he was unable, as it flowed out of the wound as fast as he swallowed it, causing violent cough and sense of suffocation.

*Let his mouth be kept moist with lemonade, but no attempt made to swallow.—Habeat enema purgans statim.*

*Vespere.* General appearance of countenance and eyes less expressive of languor, impatience, or distress. Face less pale; lips have, in a certain degree, recovered their natural hue, and extremities are of a natural warmth. Pulse 100, small and rather hard; temperature 98. Skin dry; cough and respiration as at last report; thirst urgent: had several copious stools from the enema; is now perfectly quiet, and when asked if he has much thirst, he makes signs that it is excessive.

*Let eight ounces of lemonade be injected per anum every two hours, until the thirst is mitigated.*

Dressings have been repeated, on account of the moisture passing by the wound.

*December 22d,* A. M. Has passed a very restless night, and slept none. Countenance and eyes expressive of much greater languor, anxiety, and distress, than at any former visit; face somewhat flushed, and feels rather warm to the touch; eyes rolling quickly, and at times staring; respiration very difficult and laborious; cough exceedingly painful and frequent: he makes signs that he is unable to breathe in the position in which it is necessary to keep his head. Pulse 112, small, but hard; temperature 99. Skin dry, thirst urgent, tongue foul; passes the lemonade as fast as it is thrown up.

*Let the injection of lemonade be continued, but in smaller quantity.*

On removing the dressings, the parts appeared very much apart; on which account it was thought advisable to pass a stitch on each side, through the muscular substance on the sides of the *os hyoides* and cartilage, and cover them with straps of adhesive plaister.

*Horâ 2dâ,* P. M. General appearance of countenance and eyes much as at last report; can now rest more comfortably, and breathe with more freedom. Complains much of thirst and an unpleasant gnawing sensation in the region of the stomach. Functions as at last report. Has retained the enema.

*Let a pound of milk, in the course of the day, be thrown into the stomach by means of an elastic gum catheter.*

*Vespere.* Appearance nearly as at last report; felt great relief from the milk thrown into the stomach; has retained several enemas of lemonade; thirst much abated. Other symptoms and functions as before.

*To be dressed as yesterday.*

*December 23d,* A. M. Passed a very bad night, slept none, and continued tossing about his bed the whole night, moaning greatly, and appearing to suffer much. Countenance depicts the greatest possible distress; eyes staring, red, and at times rolling quickly. He is extremely restless, tossing his head about in all directions, and finds it quite impossible to repose when the head is sunk upon the breast. Inspiration is performed with great labour of the respiratory muscles, and with the wheezing sound formerly described; it is impeded also by frequent convulsive attacks, approaching almost to suffocation. He has been rather delirious during the night, and has made frequent signs that his thirst is intolerable, and that the dressings cause great uneasiness. Pulse 112, small and rather hard; temperature 100. Skin dry; tongue covered with a thick white fur; thirst not so great: no stool since last report.

*Repeat the milk, and lemonade enema.—Let all the stitches be removed, and the wound covered with a pledget of ungt. ceræ and a bandage*; *and let the head be kept as nearly as possible in the position formerly advised.*

*Horâ 12mâ.* General appearance of countenance and eyes much worse, and all the symptoms greatly aggravated; it is now with the greatest difficulty he can draw his breath; his eyes are swollen, red, and suffused; he stares around him with a wild look of despair, almost indescribable. It is now almost impossible to introduce the flexible tube, on account of the great irritability of the glottis, every attempt causing violent convulsive cough, as well as painful retchings. Stitches have been removed, but this gave no relief. Functions as at last report.

*Continue as before,* et fiat venæsectio ad ℥xl. statim.

*Vespere.* Appearance of countenance and eyes somewhat improved: can now respire with a great deal more freedom, and rests in one position, with the head inclined a little forward; does not complain so much of thirst, and seems much gratified by the administration of the enemas. He has had no attacks of convulsive cough, or difficulty of breathing, since two hours after the bleeding; closes his eyes from time to time, and seems inclined to sleep, but suddenly starts in a fright, which causes great distress for some time afterward. Pulse 108, small, but soft; temp. 99; skin clammy; tongue white and loaded; thirst urgent; no appetite. No stool since last report.

*Repetatur enema purgans.—Continue the milk and lemonade.*

December 24th, A. M. Passed a tolerable night and slept a little, though rather disturbed. He was neither delirious, nor moaned much during the night. Countenance more composed, though still rather anxious and impatient; but the redness and suffusion of eyes were gone. No flushing of the face, nor does it feel warm to the touch.

Complains of considerable pain in the site of the maxillary glands, which are a little swelled; also of pain in the course of the wound; cough and respiration better; can rest with some degree of comfort, and makes no complaint of thirst. Sore looks well, the surface being covered with healthy florid granulations. Pulse 80, and small; temp. 98; skin moist; appetite improved; tongue white, but moist; thirst moderate; several stools from the enema.

*Continue the milk and lemonade, as before.*

December 25th, A. M. Passed a very good night, and slept quietly for two or three hours. General state much as at last visit. Swelling of the glands much increased, particularly of those at the left angle of the lower jaw. Sore looks well, but without the slightest appearance of adhesion at any point. He feels quite easy at present; had a slight fit of difficulty of breathing this morning, which lasted but a very short time. Cough easy; pulse 80, small, and soft; heat 98; skin moist; tongue clean; appetite improves; little or no thirst; bowels regular.

*Omit. enema acidul. Continuetur lac, et insuper, injiciatur in ventriculum, ope tubi flexilis, jusculi bovini* ℔i. *quotidie, necnon applic. glandulis tumefact. ol. amygdal. c̄. panno laneo.*

It seems unnecessary farther to transcribe the daily reports, as thence-forward nothing very deserving of notice took place in the course of his slow and gradual advance to recovery.

The fits of dyspnœa and convulsive cough occasionally recurred, but they were not of any comparative severity or duration, and readily yielded to the inhalation of the vapour of warm water. The general treatment consisted, as before, of the daily use of milk and beef tea, and the occasional exhibition of a laxative enema. The wound was dressed simply, twice a-day.

On the 30th of December, during the time of dressing the wound, he expressed an eager wish to attempt the swallowing of a little milk, in which I thought it right to indulge him; but, to his infinite mortification, the fluid passed out at the wound in a full stream, some of it getting into the larynx, and causing violent spasmodic cough.

By the 6th of January, considerable adhesions had taken place at the posterior and lateral parts of the wound. On the 10th of February the wound was firmly united through its whole track, and deglutition was performed with very little inconvenience; however, there remained an ulcer on the anterior part of the neck, over the thyroid cartilage, laying the latter bare to a considerable extent at its upper part. This was caused by the laceration and consequent ulceration of that portion of integuments through which the stitches had been passed, previously to his admission into the hospital. In the course of three weeks this ulcer healed, without the occurrence of any untoward symptom; and having recovered his health and strength, he was, on the 21st of March, 1814, discharged to His Majesty's ship *Berwick*, whence he came No unpleasant consequence remained from the wound save an alteration in his voice, which is now harsh and croaking.

On perusing the above details, the reader may, perhaps, ask, Why the lemonade, instead of being injected per anum, was not thrown into the stomach by the flexible tube, as well as the milk? It arose from my wish to lessen, as much as possible, the sufferings of the patient; for the introduction of the tube through which the milk was thrown, caused always violent irritation, pain, and cough. How much greater must these have been, had we passed down the tube, as often as his thirst called for the exhibition of the lemonade? Besides, I found on trial, that the thirst was very effectually alleviated by the enema every two hours. I should have preferred throwing in the milk by a tube introduced through one of the nostrils; but I found this impracticable, on account of the position of the head, which was required to be kept bent, the chin almost resting on the breast.

From the result of this case, I should scarcely, in similar instances, be induced to have recourse to stitches, as the irritation they excite in the soft parts is unavoidably propagated to the larynx and lining membrane of the trachea, thus aggravating the convulsive cough and dyspnœa, which are always the most painful and the most dangerous symptoms in such wounds. All the benefit of sutures can be secured by bandaging and retaining the head in the position above described.

The practical reader will mark with peculiar pleasure the commanding influence which the large bleeding (forty ounces being abstracted at once), had in lessening general irritation, as well as in ameliorating the local condition of the wound.

In justice to Dr. James Cowan, at that time hospital-assistant, I cannot but mention how much this patient was indebted to the zeal, assiduity, and humanity of that gentleman.

W. BOYD, M. D.

September 20, 1817.

